# High out-of-clinic blood pressure is associated with adiposity indicators in leisure physical activity practitioners in Midwest Brazil

**DOI:** 10.1186/s40885-018-0093-5

**Published:** 2018-04-23

**Authors:** Bruna M. Giglio, Renata C. Fernandes, Ana B. Marini, João F. Mota, Gustavo D. Pimentel

**Affiliations:** 1 Clinical and Sports Nutrition Research Laboratory (Labince), Nutrition Faculty (FANUT) Federal University of Goias (UFG), Goiânia, GO Brazil; 2 Gabinete 10, Faculdade de Nutrição (FANUT) Universidade Federal de Goiás (UFG), Rua 227, Quadra 68 s/n°, Setor Leste Universitário, CEP: 74605-080, Goiânia, GO Brazil

**Keywords:** Hypertension, Adiposity, Metabolic diseases, Exercise

## Abstract

**Background:**

Several diseases, such as obesity, hypertension and type 2 diabetes are frequently associated with metabolic abnormalities, high costs of healthcare and morbi-mortality; thus the aim of this study was to investigate the relationship between out-of-clinic high blood pressure and chronic disease-associated adiposity indicators in practitioners of leisure physical activity.

**Methods:**

A cross-sectional study with 414 subjects of both genders aged 24–65 years. Data were collected by trained interviewers in five public parks. Body Mass Index (BMI), triceps skinfold and waist circumference (WC) were evaluated. Exercise training, smoking status, alcohol consumption and hypertension diagnosis were self-reported. Casual glycemia concentrations were collected and blood pressure was measured out-of-clinic once during the study. Participants with systolic **≥**140 mmHg and diastolic **≥**90 mmHg blood pressures were classified as high blood pressure. All analyses were adjusted for age and sex.

**Results:**

High-blood pressure was diagnosed in 31.4% (*n* = 130), but 34 (8.3%) from patients took medication anti-hypertensive and were previously hypertension diagnosed. Participants with high blood pressure had a higher BMI (25.66 vs. 26.87 kg/m^2^; *p* = 0.012), WC (90.92 vs. 95.02 cm; *p* = 0.001), and systolic and diastolic blood pressure (*p* <  0.0001) when compared to subjects with normal blood pressure. Logistic regression analysis revealed that overweight status assessed by BMI, triceps skinfold and WC increases the high blood pressure probability by approximately 1.61 (95 CI%: 1.06–2.45), 1.02 (95%CI: 1.01–1.05) and 1.61 (95%CI: 1.06–2.45), respectively.

**Conclusions:**

Adiposity indicators are associated with high out-of-clinic blood pressure measured in practitioners of leisure physical activity.

## Background

The World Health Organization estimated 56 million deaths worldwide in 2012, out of which 38 million were due to chronic noncommunicable diseases (NCDs). Noncommunicable diseases are increasing and may be responsible for 52 million deaths worldwide in 2030; in Brazil, the proportion may reach 74% of all deaths. Obesity, hypertension and type 2 diabetes mellitus are factors that contribute to the high rate of NCDs’ morbidity and mortality. However, leisure physical activity helps in the prevention and treatment of hypertension, and reduces cardiovascular risk and mortality [1–3]. Considering the effects of high blood pressure and low leisure physical activity on health, diagnosing hypertension is of supreme importance.

Out-of-clinic blood pressure monitoring is increasingly regarded as a routine component of the management of the odds of high blood pressure. It enables early detection of high blood pressure in patients with NCDs, facilitating treatment and reducing the risk factors. The diagnosis of hypertension, based on out-of-clinic blood pressure monitoring, can be useful to assist the patient in the context of primary care in low- and middle-income countries. In addition, out-of-clinic blood pressure monitoring offers an earlier estimate of NCD complications compared to clinic blood pressure measurement [4, 5]. Thus, we aimed to investigate the relationship between out-of-clinic high blood pressure and chronic disease-associated adiposity indicators in practitioners of leisure physical activity in public parks.

## Methods

### Study design and sample

A cross-sectional study was conducted from May to July 2016 in participants recruited in five different public parks. The sample consisted of 414 adults and elderly of both genders. The sample size was calculated for Power analysis [6] considering n = PxQ/(E/1,96)^2^, where: *n* = minimum sample required, P = maximum prevalence of hypertension in the city of Nova Andradina, State of Mato Grosso do Sul, which is 21.7% for adults and elderly of both sexes over 39 years of age [7], Q = 100 – P, E = acceptable margin of error. A minimum sample required was 334 patients.

The inclusion criteria were men and women who attended in the parks to do leisure physical activity, we do not restrict the participation regarding to age, nutritional status, blood pressure levels and use medication routine. In addition, none restriction was performed regarding to anti-hypertensive medication use before blood pressure was measured. The exclusion criteria adopted was if only they had presence of physical and locomotive problems and those unable to respond to the anamneses. The study was approved by the Ethics Research Committee under protocol No. 1.470.285/2016. All participants signed the informed consent form designed according to the n° 466/12 on “Research involving human beings, from the Health Board of the Ministry of Health”.

### Socioeconomic evaluation

Data were collected by trained interviewers. The self-report socioeconomic questionnaire collected demographic data (city of residence, age and education in years) and lifestyle indicators (exercise training, alcohol consumption and smoking status).

### Anthropometric measurements

Body weight was measured using a platform anthropometric scale (Filizola®), and height by a portable estadiometer (SECA®) to enable body mass index (BMI) calculation. Abdominal obesity was determined by waist circumference (WC) using an inelastic tape measured with the patient standing following the recommendations of guidelines of the European Society of Hypertension [8]. The triceps skinfold thickness was measured in triplicate at the midpoint of the upper arm using the Lange® skinfold caliper. Trained nutritionists performed all the measurements.

### Evaluation of the physical exercise

Physical exercise was assessed by self-report. We asked about the frequency, intensity and type of physical activity. Participants who did not exercise were classified as sedentary.

### Blood pressure and casual glycemia measurement

Blood pressure was taken once, after all the other measurements to ensure the patient had rested for eight to 10 min. A calibrated automatic digital wrist blood pressure monitor (Omron, model HEM-637®) was used according to the manufacturer’s instructions. This equipment has been used previously [9, 10] and validated [11–13]. Although the wrist blood pressure monitor use is not recommended, it use may be justified for excess body weight subjects [8].

Blood pressure was measured with participants in the seated position, feet flat on the floor and left arm relaxed (resting on the table or on the right arm) at heart height with the palm of the hand facing the chest. Participants were required to remain silent during measurements. The high blood pressure classification (systolic blood pressure [SBP] ≥160 mmHg or diastolic blood pressure [DBP] ≥100 mmHg) were considered as diagnostic criteria, according to values of blood pressure of the European Society of Hypertension [10].

Casual glycemia was determinated in blood obtained of hand finger puncture and measured using the portable monitor and reagent strips (Roche®).

### Statistical analysis

Analyses were performed using the MedCalc® program. Data were described as means and standard deviations and the Kolmogorov-Smirnov test was applied to test normality. To compare the groups with normal and high blood pressure, we used the independent t-test. To verify the association between men and women’s smoking status and alcohol intake among those with normal or high blood pressure, we performed the Fischer exact test.

Logistic regression was used to calculate the odds ratio (95%-CI) of patients with high systolic blood pressure [SBP] ≥160 mmHg or diastolic blood pressure [DBP] ≥100 mmHg [10]. The regression analysis logistics were performed unadjusted (crude model) and adjusted for sex and age (model 1). To correlate the indicators BMI, triceps skinfold thickness and WC with the systolic and diastolic blood pressure, the Pearson’s correlation test was performed. The statistical significance level was set at *p* <  0.05.

## Results

A total of 414 practitioners of leisure physical activity were evaluated, comprising 195 men and 219 women, with a mean age of 45.2 years. Participants’ data were divided into normal (*n* = 284, 68.6%) or high (*n* = 130, 31.4%) blood pressure groups (Table 1). From individuals with high (*n* = 130) blood pressure, 34 (8.3%) patients took medication anti-hypertensive and were previously hypertension diagnosed by physician and self-reported by patients, and 96 (23.2%) had no hypertension diagnosed.

**Table 1:** Characteristics of screening in Midwestern Brazilian participants

Characteristics	Normal blood pressure *n* = 284 (68.6%)	High blood pressure *n* = 130 (31.4%)	*P value*
Age (y)	44.57 ± 17.16	46.45 ± 15.59	0.312
Sex (%)			0.272^1^
Male	121 (29.2)	74 (17.8)	
Female	153 (39.5)	56 (13.5)	
Smoking status (%)			0.253^1^
No	266 (64.2)	114 (27.5)	
Yes	18 (4.4)	16 (3.9)	
Alcohol intake (%)			0.518^1^
No	152 (36.8)	114 (14.2)	
Yes	132 (31.9)	71 (17.1)	
Exercise frequency (time/wk)	1.62 ± 1.35	1.60 ± 1.39	0.890
Exercise training (minutes/wk)	70.75 ± 34.81	64.83 ± 24.15	0.152
BMI (kg/m^2^)	25.66 ± 4.24	26.87 ± 5.04	0.012*
Triceps skinfold (mm)	23.07 ± 7.99	24.00 ± 10.24	0.322
Waist circumference (cm)	90.92 ± 11.18	95.02 ± 12.71	0.001*
Casual glycemia (mg/dL)	104.74 ± 34.84	107.72 ± 35.67	0.425
Systolic blood pressure (mmHg)	124.75 ± 15.16	141.05 ± 21.65	< 0.0001*
Diastolic blood pressure (mmHg)	84.19 ± 10.54	99.51 ± 20.38	< 0.0001*

^1^2 × 2 table, Fischer’s exact test. BMI: body mass index

^*^test t’s Student

Table 1 shows the profile of individuals stratified by sociodemographic and clinical data. The prevalence of smokers, alcohol intake and frequency and time of exercise did not differ between groups. However, the BMI (*p* = 0.012), waist circumference (*p* = 0.001), systolic (*p* <  0.0001) and diastolic (p <  0.0001) blood pressure were greater in the high than normal blood pressure group.

Next, we performed a logistic regression analysis (Table 2) to discover any associations between the anthropometric markers (BMI, triceps skinfold or WC) and a higher probability of increased blood pressure. Thus, BMI was dichotomized as raised (overweight or obese), ≥25 kg/m^2^ for adult (World Health Organization) and ≥ 27 kg/m^2^ for elderly (≥60 years) [14]. A raised BMI was found to increase the high blood pressure odds 1.61 (95%CI: 1.06–2.45) compared to individuals with low BMI in a crude model, but no after adjusting for sex and age. Regarding the triceps skinfold measurement, those > 90th percentile was not associated with high blood pressure odds in the crude model, but was associated after adjusting for sex and age 1.02 (95%CI: 1.01–1.05). A high WC (≥102 cm for men and ≥ 88 cm for women) was associated with high blood pressure odds of 1.61 (95%CI: 1.06–2.45) in the crude model, but no after adjusting for sex and age.

**Table 2:** Logistic regression for higher blood pressure levels (Systolic: ≥160 or Diastolic: ≥100 mmHg) in accordance with alcohol consumption, smoking status and anthropometric indicators in Midwestern Brazilian subjects

Indicators	OR (95%IC) Crude	*P value*	OR (95%IC) Model 1^1^	*P value*
Alcohol (No/Yes)	1.38 (0.91–2.10)	0.124	1.25 (0.82–1.92)	0.288
Smoking status (No/Yes)	2.07 (1.02–4.21)	0.043*	1.86 (0.91–3.82)	0.088
Exercise (No/Yes)	1.01 (0.70–1.46)	0.925	1.01 (0.69–1.46)	0.954
BMI (Adult: ≥25 or Elderly: ≥27 kg/m^2^)	1.61 (1.06–2.45)	0.024*	1.46 (0.95–2.26)	0.081
Triceps skinfold (Percentil > 90)	1.01 (0.98–1.03)	0.322	1.02 (1.01–1.05)	0.045*
WC (♂: ≥102 cm and ♀: ≥88 cm)	1.61 (1.06–2.45)	0.025*	1.18 (0.64–2.14)	0.586
Casual glycemia (≥ 200 mg/dL)	1.08 (0.26–4.39)	0.911	1.00 (0.24–4.13)	0.998

*BMI* Body mass index, *OR* Odds Ratio, *WC* Waist circumference

^1^Model 1: adjusted by sex and age

In addition, smoking status was associated in 2.07 (95%CI: 1.02–4.21) times with high blood pressure in crude model, but no after adjusting for sex and age (Table 2). Alcohol consumption, physical exercise and casual glycemia concentrations were not associated with odds of high blood pressure levels (Table 2).

**Fig. 1:**
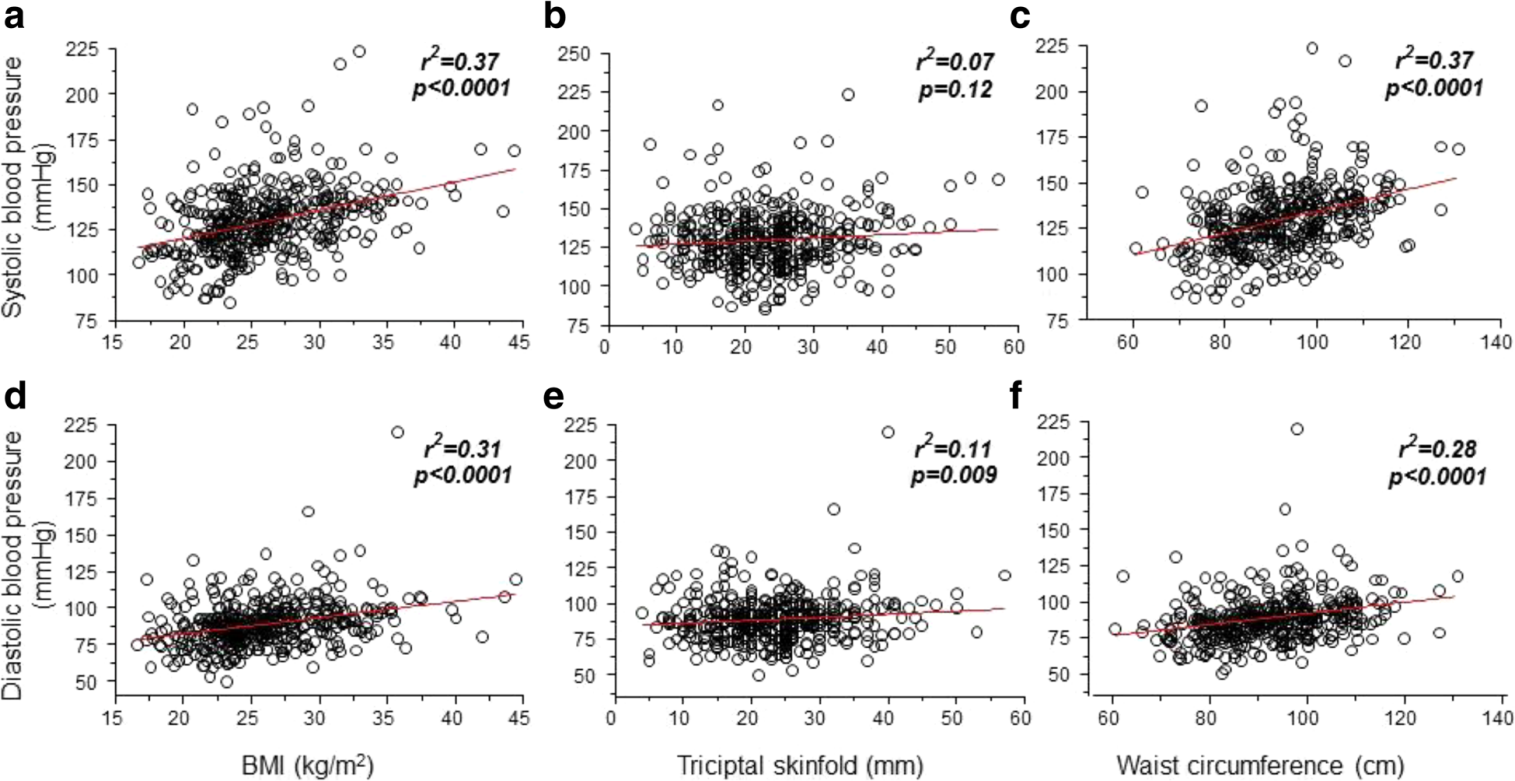
Correlation between diastolic and systolic blood pressure with anthropometric indicators. BMI: body mass index

The Pearson’s correlation analysis revealed that both diastolic and systolic blood pressures were associated with anthropometric indicators (Fig. 1). In addition, the BMI and WC were positively correlated with systolic and diastolic blood pressure. The triceps skinfold measurement was positively correlated only with the diastolic, but not with the systolic blood pressure (Fig. 1).

## Discussion

In this study, we found out-of-clinic measurements of high blood pressure, and that adiposity is associated this. Moreover, we demonstrated that people who use the public parks presents several risk factors for the development of high blood pressure. Another important finding was the increased diastolic and systolic blood pressure levels, which were positively associated with a higher BMI, triceps skinfold and WC.

We performed a logistic regression analysis to obtain the precise relevance of each anthropometric measurement and socioeconomic factor as a high blood pressure probability marker. Individuals with a higher BMI, triceps skinfold and WC measurement also had raised brachial arterial pressure, which remained after adjusting for sex and age. Warrer et al. [15] revealed that a raised WC increases by five times the risk of developing hypertension and diabetes. Additionally, an increase in BMI was found to lead to a 30% increase risk of becoming hypertensive compared to subjects who had no change in body weight [16].

Although the individuals carried out leisure physical activity, such as walking and jogging, and racing in the parks, they presented high out-of-clinic blood pressures (*n* = 130, 31.4% of individuals), leading us to suggest that their time, intensity and frequency of exercise were low and that the adiposity indicators, such as BMI, triceps and WC and smoke status were responsible for raising their blood pressure levels. On the other hand, we have demonstrated previously that nutritional counseling can reduce adiposity indicators and to prevent NCDs [17, 18].

We can speculate that monitoring out-of-clinic blood pressure in, for example, public parks can be very important as it helps to control adiposity and prevent increased blood pressure levels. Although the exercises performed in public parks are protect to some extent against NCDs it seems that, for the control of high blood pressure, they do not. Therefore, more studies are needed to verify the appropriate physical activity in terms of its intensity and duration to prevent high blood pressure.

A limitation of this study is that we evaluated only Midwestern Brazilian subjects, but though is not a representative sample of other regions of Brazil, the sample size calculus revealed that we evaluated more individuals than the sample size. Although we used only the wrist blood pressure monitor, this equipment has been validated and adequate for overweight people. And even though the blood pressure was measured only once, all individuals were asked to remain seated when completing the socioeconomic questionnaire to avoid changes in their blood pressure levels and so the blood pressure was measured after eight to 10 min at rest.

## Conclusions

In summary, we found that high out-of-clinic blood pressure is associated with chronic disease-linked adiposity indicators in leisure activity practitioners in Midwest Brazil. This information is useful for developing nutritional and physical activity interventions in primary care to attenuate co-morbidities and chronic disease-related metabolic consequences.

## Abbreviations

BMI: Body mass index
NCDs: Chronic noncommunicable diseases
WC: Waist circumference
